# The complete mitochondrial genome of *Menticirrhus littoralis* (Sciaenidae, Perciformes) and its phylogeny

**DOI:** 10.1080/23802359.2020.1772681

**Published:** 2020-06-02

**Authors:** Adrianne Freitas, Jeferson Carneiro, Aurycéia Guimarães-Costa, Horacio Schneider, Iracilda Sampaio

**Affiliations:** a Genomics and Systems Biology Center, Universidade Federal do Pará, Belém, Brazil; b Institute of Coastal Studies, Universidade Federal do Pará, Bragança, Brazil

**Keywords:** mtDNA, phylogeny, *M. littoralis*, fish, Perciformes

## Abstract

We describe the mitochondrial genome of the *Menticirrhus littoralis* and infer the phylogenetic position of *Menticirrhus* in the family Sciaenidae. The genome contains 16,499 base pairs distributed in gene regions (13 protein-coding regions, 2 rRNAs and 22 tRNAs) and a control region (CR). Our phylogenetic analysis suggests, with strong statistical support, that *Menticirrhus* is a sister group of the other sciaenids.

The gulf kingfish, *Menticirrhus littoralis* is widely distributed in the Western Atlantic, from the USA to Uruguay (Márquez et al. 2013). They are common in shallow and frequent waters as companion fauna in shrimp fishing ([Bibr CIT0007][Bibr CIT0007]). Previous research has been unable to define satisfactorily the phylogenetic position of the genus *Menticirrhus* in the family Sciaenidae (Chao 1978; Sasaki 1989; Santos et al. 2013; Lo et al. 2015), what is one of the objectives of this work besides the description of the complete mitochondrial genome of the species.

One individual of *M. littoralis* collected in the Caeté River estuary (0 49′10″ S 46 36′30″ W) was used for genome sequencing and was deposited in the ichthyological collection of the Museu Paraense Emílio Goeldi (MPEG), under voucher number MPEG 38936.

For DNA extraction was used the commercial Wizard® Genomic DNA purification kit (Promega). We developed 20 primers pairs for the PCR, and we sequenced the samples by Sanger et al. (1977). The complete sequence was annotated in the MitoAnnotator (Iwasaki et al. 2013), and reevaluated the tRNAs in tRNAscan-SE1.21 (Lowe and Eddy 1997). The phylogenetic tree was based on the Maximum Likelihood in RAxMLGUI (Silvestro and Michalak 2012) using 13 PCGs and two rRNA genes.

The mitochondrial genome of *M. littoralis* (GenBank accession number: MT199157) has a total of 16,499 base pairs (bps), arranged in two rRNAs (12S and 82 16S), 13 protein-coding genes, 22 tRNAs, and a Control Region. The nucleotide composition of the adenine is 26.3%, 32% cytosine, 16.9% guanine and 24.8% thymine.The size, structural organization, and location of the different components of the mitogenome are consistent with those of other organisms (Anderson et al. 1981).

The start codon of all coding regions is ATG, that is, the amino acid Methionine, a variety of stop codons, and in many cases are incomplete stop codon T or TA, which can presumably be completed to TAA via post-transcriptional polyadenylation (Ojala et al. 1981).

As in other vertebrates, the 12S and 16S ribosomal genes are located between tRNA-Phe and tRNA-Leu (UUR), and all 22 tRNAs can be folded into typical cloverleaf secondary structures, except for tRNA-Ser (AGY), which lacks the DHU stem.

The Control Region has a total length of 824 bps, and has a higher than average A + T content (62.2%) in comparison with the mitogenome as a whole (51.1%), which found to be a characteristic of the Percoidei (Xu et al. 2011).

The phylogenetics result of present study placed *M. littoralis* as a sister group of all the other sciaenids analyzed, with strong statistical support (Figure 1). This finding provide new insights into the phylogenetic position of *Menticirrhus* within the family Sciaenidae, in addition to contributing to the understanding of the evolutionary history of the sciaenids through the sequencing of fish mitogenomes.

**Figure 1.**
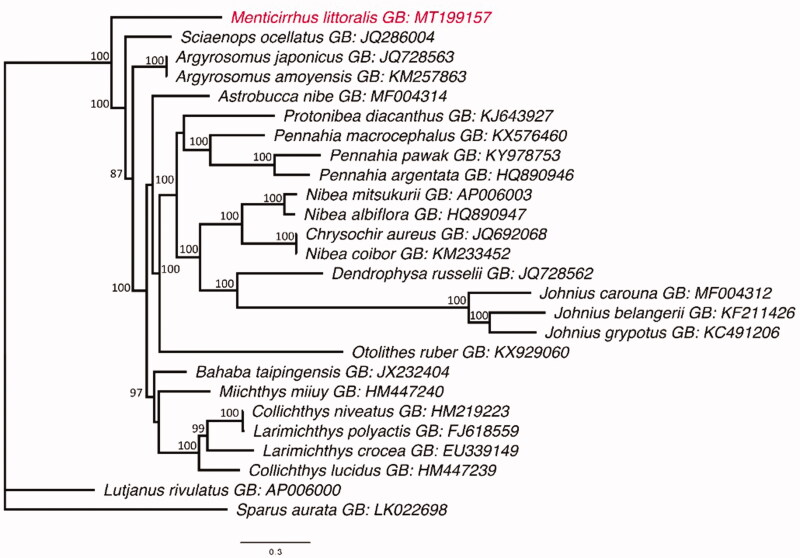
The Maximum Likelihood topology of the Sciaenidae, based on the mitogenome, showing the phylogenetic position of *M. littoralis* (in red). Numbers at nodes represent bootstrap values in percentage. Values below 70% are not shown. The tree is rooted with *Lutjanus rivulatus* and *Sparus aurata*. GB is the GenBank Accession Number.

## Data Availability

The whole mitochondrial genome are available at NCBI with the GenBank accession no. MT199157.
